# Program of Seven 45-min Dry Immersion Sessions Improves Choice Reaction Time in Parkinson’s Disease

**DOI:** 10.3389/fphys.2020.621198

**Published:** 2021-01-14

**Authors:** Alexander Yu. Meigal, Olesya G. Tretjakova, Liudmila I. Gerasimova-Meigal, Irina V. Sayenko

**Affiliations:** ^1^Laboratory of Novel Methods in Physiology, Institute of Higher Biomedical Technologies, Petrozavodsk State University, Petrozavodsk, Russia; ^2^State Scientific Center, “Institute of Biomedical Problems,” Russian Academy of Sciences, Moscow, Russia

**Keywords:** simple reaction time, choice reaction time, discriminative reaction time, Parkinson’s disease, “dry” immersion, microgravity, tapping test

## Abstract

The study hypothesis held that in subjects with Parkinson’s disease (PD), the reaction time (RT) tests of the higher cognition demand would have more readily improved under the program of analog microgravity (μG) modeled with “dry” immersion (DI). To test this hypothesis, 10 subjects with PD have passed through a program of seven DI sessions (each 45 min long) within 25–30 days, with overall μG dose 5 1/4 h. Five patients were enrolled as controls, without DI (noDI group). Simple RT (SRT), disjunctive RT (DRT), and choice RT (CRT) were assessed in four study points: before the DI program (preDI), 1 day after the DI program (postDI), 2 weeks after the DI program (DI2w), and 2 months after the DI program (DI2m). The motor time (MT) was assessed with the tapping test (TT). Additionally, signal detection time (SDT) and central processing time (CPT) were extracted from the data. Before the program of DI, the RT tests are in accordance with their cognition load: SRT (284 ± 37 ms), DRT (338 ± 38 ms), and CRT (540 ± 156 ms). In accordance with the hypothesis, CRT and DRT have improved under DI by, respectively, 20 and 8% at the study point “DI2w,” whereas SRT, SDT, and MT did not change (<5% in the preDI point, *p* > 0.05). Thus, the program of DI provoked RT improvement specifically in the cognitively loaded tasks, in a “dose of cognition-reaction” manner. The accuracy of reaction has changed in none of the RT tests. The neurophysiologic, hormonal/neuroendocrine, behavioral, neural plasticity, and acclimation mechanisms may have contributed to such a result.

## Introduction

Parkinson’s disease (PD) is increasingly being studied due to its growing global prevalence (Wirdefeldt et al., 2011), high economical burden on society (Martinez-Martin et al., 2019), and negative impact on the quality of life (Marras et al., 2008). Gradually, PD becomes a kind of “model” disease for testing novel approaches in diagnostics, treatment, and rehabilitation (Pasluosta et al., 2015).

Besides specific motor (rest tremor, muscle rigidity, bradykinesia, balance, and gait impairment) and non-motor (cognitive and emotional and symptoms) (Rodriguez-Oroz et al., 2009), PD is characterized by some not readily seen signs, such as impaired perception of time, increased reaction time (RT) to stimuli (Yabe et al., 2019), and general slowdown of cognition (Vlagsma et al., 2016). These subtle deficits can seriously deteriorate the daily life activities of PD patients, since RT and accuracy of reaction are of vital importance when, e.g., driving a car or crossing the street (Block and Gruber, 2014). Evaluation of RT is widely used as a technically feasible and informative indicator of the neurophysiologic processes in the brain (MacDonald and Meck, 2004; Lindenberger, 2014; Criaud et al., 2016).

Among the most commonly used RT tasks are simple RT (SRT) and choice RT (CRT). In the SRT task, a subject reacts with motion on a visual or audio stimulus as fast as possible. In young subjects, SRT lasts 200–220 ms; in older subjects, it is extended to 230–250 ms (Yordanova et al., 2004; Woods et al., 2015b; Tokushige et al., 2018; Otaki and Shibata, 2019); and in PD patients, it is 300–360 ms (Jordan et al., 1992; Müller et al., 2001; Tokushige et al., 2018). Longer SRT correlates with increased degree of depression (Cooper et al., 1994) and slower time processing (Tokushige et al., 2018). Neurophysiologically, SRT corresponds to early cortical components of the evoked brain potential (Yordanova et al., 2004) and perceptual and motor stages of signal processing (Cooper et al., 1994).

Unlike SRT, CRT evaluates the minimal time needed to choose between two, or more, stimuli. Therefore, CRT allows evaluating far more complex cognition processes, e.g., recognition, categorization/sorting, and decision-making, which activate specific nervous structures including the basal ganglia, thalamus, and cortex (Dunovan et al., 2019). The more complex the CRT task is, the longer the RT is and the more circuits of the brain it recruits (Hare et al., 2011; Hunt et al., 2018). In young controls, CRT varies from 300 to 470 ms, which may correspond to the cognitive component (P300) of the evoked brain potential (Woods et al., 2015a; Otaki and Shibata, 2019). Furthermore, CRT depends on the level of such brain mediators involved in decision-making as dopamine and norepinephrine (Yordanova et al., 2004; Hassall et al., 2019). CRT is extended to 500–600 ms in the elderly (Woods et al., 2015a) and to 600–1000 ms in PD patients (Cooper et al., 1994).

There is a variant of the RT task, in which one should respond to given and not respond to other, also given, stimuli within a “go/no-go” paradigm, referred to as the disjunctive, or discriminative, RT task (DRT) (Redfern et al., 2017). The DRT task allows evaluating attention/vigilance and the function of the additional motor cortex involved in the inhibition of unwanted movement and the activation of the desired movement (Criaud et al., 2016).

The SRT tasks require advanced preprogramming, whereas the CRT tasks rely on online processing (Maslovat et al., 2014). The SRT and CRT tasks are clearly differentially loaded with cognition as manifested in their different RTs and different responses to aging and therapies. The more complex the RT task is, the greater the RT slowing across the life span is (Fozard et al., 1994). In PD patients, CRT is selectively improved by the transcranial current stimulation (Elder et al., 2016), deep brain stimulation (Georgiev et al., 2016), and levodopa intake (Müller and Harati, 2020). However, there is evidence that in patients with PD, levodopa and deep brain stimulation improve SRT rather than CRT (Temel et al., 2006).

Both SRT and CRT are connected to neurophysiological mechanisms involved in the perception of time flow or timing (MacDonald and Meck, 2004). In young subjects, timing is optimal (Yabe et al., 2019), but in aging persons, it is slightly impaired, though in a milder form compared with PD patients (Dykiert et al., 2012). In PD patients, deterioration of perceiving the event and violation of the correct interval timing can lead to a general slowdown of the thought processes, or “bradyphrenia,” bradykinesia (Vlagsma et al., 2016), and, presumably, increased RT. Thus, the RT tasks of differential complexity seem reliable for testing the effectiveness of anti-PD therapies.

Varied rehabilitation programs for PD based on exercises (Flach et al., 2017; Combs-Miller and Moore, 2019), music (Hove and Keller, 2015), dance (Dos Santos Delabary et al., 2018), Tai Chi (Liu et al., 2019), Yoga practices (Green et al., 2019), and virtual reality (Asakawa et al., 2019) exerted beneficial clinical effect on PD. However, this effect was very similar among these anti-PD therapies and was short-term (Tomlinson et al., 2012, 2014).

A program of analog microgravity (μG) modeled with “dry” immersion (DI) was recently shown to reduce the scores of muscle rigidity and depression in PD (Meigal et al., 2018). Furthermore, the regularity of the surface electromyogram, indicative of tremor reduction, was decreased after a single short DI session (Miroshnichenko et al., 2018). Programs of common water immersion combined with exercises exerted similar beneficial effect on cognition and motion in elderly people without PD (Kim et al., 2018), presumably due to more efficient cortical processing of somatosensory signals (Sato et al., 2012). In healthy subjects, during the condition of 0 gravity induced with parabolic flight, RT has decreased specifically in a more complex task (Wollseiffen et al., 2016, 2019).

In sum, these data allow hypothesizing that in subjects with PD, analog μG would have decreased RT *via* either decreased rigidity (faster contraction speed) or improved mental processing. Furthermore, the RT tasks with higher cognition load would have been more readily modified under DI. To check these research questions, we challenged varied RT tests in subjects with PD with a program of DI. Among the RT tests, we chose CRT, DRT, and SRT as they represent a spectrum of tasks with degrading cognition load.

## Materials and Methods

### Subjects

Thirteen subjects with PD were enrolled in the study. Eight of them have passed through the program of DI (DI group). Three subjects were examined as the control group (noDI group). Two subjects participated in both groups, first in the noDI and then, 1 month later, in the DI group. The two crossover subjects (9 and 10) had almost the same baseline data in both groups. Subjects 11–13 from the noDI groups had contradictions to participate in the DI group (heart arrhythmia, arterial hypertension, and convulsions in anamnesis). The anthropological and clinical data on the subjects are presented in Table 1. The general inclusion criterion was the verified diagnosis of PD. One subject had vascular parkinsonism. The non-inclusion criteria for the DI group included a variety of pathologies that potentially could have worsened under DI, e.g., epilepsy, administration of muscle relaxants, hypovolemia, atrial fibrillation, hemorrhage of various etiologies, lung diseases in the acute stage, myocardial infarction, oncologic problems, and blood clotting disorders (e.g., phlebothrombosis or thrombophlebitis) (Tomilovskaya et al., 2019).

**TABLE 1:** The anthropologic and clinical data on the subjects with PD.

No. of subjects	Age (years) and gender	Group	Height (cm), weight (kg)	Disease duration (years)	UPDRS-III scores	Stage by H and Y	Clinical form	LED* (mg/day)
1	70M	DI	176, 74	2	37	3	PD, T	187.5
2	68M	DI	167, 78	3	26	2	PD, AR	375
3	47M	DI	182, 81	3	15	1	PD, AR	No medication
4	58M	DI	170, 60	N/A	29	3	PD, T	675
5	69M	DI	179, 69	4	25	3	PD, T	250
6	65F	DI	152, 78	7	13	1	VP, AR	1,100
7	61M	DI	188, 83	6	15	1	PD, T	312.5
8	58F	DI	158, 65	N/A	26	2	PD, T	Piribedil 150 mg
9	50Ì	DI, noDI	171, 94	4	20 (DI) 21 (noDI)	2	PD, T	450
10	55Ì	DI, noDI	178, 86	6	28 (DI) 26 (noDI)	2	PD, T	450
11	46Ì	noDI	186, 114	3	15	1.5	PD, T	550
12	65Ì	noDI	164, 59	7	33	2	PD, T	850
13	69Ì	noDI	173, 93	4	33	2	PD, T	1,062.5
DI	8M, 2F	10	172 ± 11, 77 ± 10.0	4.4 ± 1.8	23.4 ± 7.5	2.0 ± 0.8	–	439 ± 284
noDI	5M	5	174.4 ± 8.4, 89 ± 19.8	4.8 ± 1.6	25.8 ± 8.0	1.9 ± 0.2	–	575 ± 189

* LED (levodopa equivalent dose) was calculated using the formula of Nutt et al. (2003). T, tremulous form; AR, akinetic–rigid form of PD; VP, vascular parkinsonism.

### Outcome Measures

#### Clinical Outcome Measures

For primary clinical outcome measures, we applied the Unified Parkinson’s Disease Rating Scale (motor part, UPDRS-III) and its subtotals related to tremor (items 20–21), muscle rigidity (item 22), and akinesia (items 23–27). The clinical examination was performed by a qualified neurologist.

#### The RT Tasks

All the RT tasks appeared as the serial reaction tasks. They were performed with PC-based NS-Psychotest (Neurosoft Ltd., Ivanovo, Russia), in a key-pressing paradigm, with operation panel (130 × 68 × 19 mm). The distance between two buttons on it was 68 mm, and press run of buttons was 0.5 mm. The subjects were allowed to familiarize the experimental setup and to train with each RT task for several minutes. To better cope with tremor at the time of RT performance, they were allowed holding the operating panel comfortable on the knees or on the table. Furthermore, the subjects were allowed to use the most effective way of pressing the button on the panel—with the thumb or the index finger. In all further trials and tasks, the subjects used the same mode of operating the panel. After training, the subjects performed two trials of the same RT task in a row with a 2-min time-out between trials. The best by time trial was taken for further analysis. No specific instructions to accord importance on speed or accuracy were provided.

#### SRT

In the SRT task, the subjects pressed one of two buttons on the control panel at fastest in response to a stimulus (red-colored round flash, 11 mm in diameter, lasting for 1 s, in the middle of the panel). In that task, either of these two buttons was “good.” The SRT task consisted of 30 serial trials within a period of 50 s. The imperative signal (flash) was presented without alert signal, in random order, with intersignal interval 0.5–2.5 s. Mean RT and accuracy rate (ratio of correct responses) were taken for analysis. Additionally, individual signal detection time (SDT), or the time needed to perceive the stimulus, was calculated by subtracting from SRT the motor time (MT) obtained from the tapping test (TT) (Woods et al., 2015b).

#### CRT

The CRT task consisted of 20 serial trials within a period of 50 s, in which the subjects had to correctly choose a stimulus (red- or green-colored flash) that appeared in the middle of the control panel by pressing at fastest the left (red-colored) or right (green-colored) button according to the color of the button. The flashes of both colors were presented without alert signal, in random order, with intersignal interval 0.5–2.5 s. The individual central processing time (CPT) was calculated by subtraction of SRT from CRT (Woods et al., 2015a).

#### DRT

**FIGURE 1:**
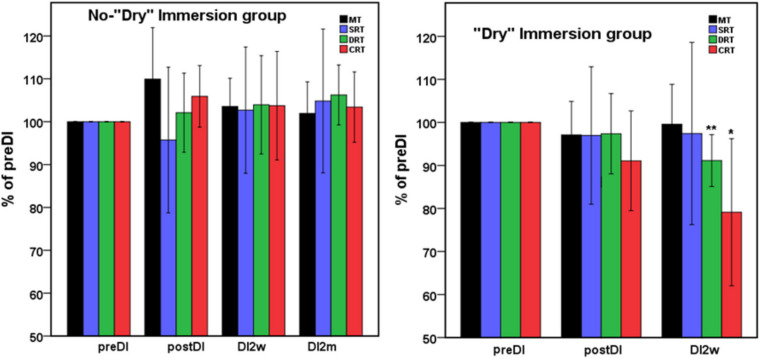
Mean percent of the studied RT tasks across the program of DI with respect to the background data (point preDI) in the control group (left) and in the study group (with DI) (right). The point “DI2m” is not presented for the DI group (right) as data on four subjects were missing. MT, motor time; SRT, simple reaction time; DRT, disjunctive reaction time; CRT, choice reaction time. Data are mean ± SD, *p < 0.05 vs. preDI, **p < 0.01 vs. preDI.

In the DRT task, the subjects responded at fastest by pressing the button on the panel on random appearance of a red circle (20 mm in diameter) in the center of the LCD screen with a white background, under a red cross (20 mm in diameter). The subjects sat 0.7 m from the PC monitor. During testing, hindrance signals in the form of circles of varied colors and sizes (12–75 mm in diameter) appeared in random places of the screen at a rate of 5 s^–1^, in a row, including the place under the cross assigned for the red circle (Figure 1). These distracting signals appeared as stop-signals and had to be ignored. The imperative signal (red circle under a red cross) was presented 30 times within 40 s. Thus, the DRT task in this study largely lays within a “go/no-go” paradigm (Criaud et al., 2016). The central feature processing time (CFPT) was computed by subtracting SRT from DRT (Woods et al., 2015a). Instrumentally, DRT was similar to the psychomotor vigilance task (Zhang et al., 2019).

#### TT

To execute the TT, the subjects tapped their wrist as quickly as possible on a PC-based contact board (82 × 58 mm), positioned on the table, with contact pencil, similar to the study of Müller and Muhlack (2010). The contact board was fixed on one place with a spare hand. Elbows were resting on the table. The TT was executed with the dominant, usually right, hand. Peak height reached by the pencil was not recorded, but it was between 1 and 3 cm. The TT task was evaluated by the total amount of contacts of the pencil and board within 30 s (N). Additionally, MT, or the time needed for one wrist flexion, was estimated by dividing the period of 30 s by doubled N (formula: MT = 30,000 ms/2 × N). Dividing by “2” was done to exclude the time of wrist rising (extension) during tapping, as flexion and extension times were considered to be equal.

### The DI Intervention

#### The DI Procedure

The condition of DI was induced by means of the “medical facility of artificial weightlessness” (MEDSIM, Center for Aerospace Medicine and Technology, State Scientific Center of Russian Federation “Institute of Biomedical Problems,” Moscow, Russia) housed in Petrozavodsk State University (Petrozavodsk, Russia). This facility has as a bathtub filled with 2 m^3^ of fresh water set at 32°C, automatically filtrated and aerated to prevent contamination, and covered with a thin waterproof material (3 × 4 m). Before DI session, the subjects laid supine for 5–7 min on a movable motor-driven platform of MEDSIM, set in the upper position above the water level, on an individual cotton sheet. During this period, electrodes for electrocardiography (ECG) and a cuff for brachial blood pressure (BP) were attached. Then, ECG was monitored in the standard lead II to search for rhythm disorders. The subjects were allowed to enter DI only if their BP did not exceed 140/80 mm Hg. After that, the subjects were wrapped in a cotton sheet and then in a waterproof material, and the platform was driven to its bottom position. As the result, the subject found her/himself immersed in water, except for the head and upper chest, without direct contact with water. The subjects were allowed to either cross their arms on the chest or hold them along the body. The DI session lasted for 45 min, and BP was monitored at the 15, 30, and 45th min (Gerasimova-Meigal and Meigal, 2019). One day prior to the study, the subjects underwent a 15-min DI trial to identify hemodynamic changes during immersion and to familiarize the procedure.

The DI session was carried out “on medication,” starting at 9:30 A.M. To synchronize the effects of anti-PD therapy with that of DI, the subjects took their medicines 2 h before the study, at 7:30 A.M. Because of the strong diuretic effect of DI, the subjects were instructed to drink 200 ml of water and urinate before DI (Tomilovskaya et al., 2019). The DI was obligatorily interrupted in case of a significant change in BP (in both directions) or at the request of the subject. After the DI session, the subjects laid motionless on the platform in its upper position for further 5–7 min for ECG recording and re-adaptation to pre-DI condition.

#### The DI Program and Study Points

The program of DI comprised seven single DI sessions, conducted twice a week (total DI dose 5 1/4 h), within 25–30 days (every 3–4 days). The data were collected at four study points: (1) before the DI program (preDI), (2) next day after the DI program (postDI), (3) 2 weeks after the DI program (DI2w), and (4) 2 months after the DI program (DI2m). The subjects were allowed to quit the program at any study point. Four subjects of the DI group were not available for the follow-up examination at the point “DI2m.”

### Statistical Analysis

The analysis was executed with IBM SPSS Statistics 21.0 (SPSS, IBM Corp., Chicago, IL, United States). Data are presented as mean ± SD. The variables were preliminarily tested for normality using the Shapiro–Wilk test. It has been found that most of the variables were not normally distributed. Therefore, the SPSS Friedman test with further *post hoc* comparisons (the Newman–Keuls test) was applied to find the difference between the RT variables along the study points. In the DI group, the study point “DI2m” was further excluded from the analysis because four individual data out of 10 were missing. To evaluate the correlation between the initial values of RT in different tasks and % of their change along the DI program, we used the Pearson’s criterion.

## Results

**FIGURE 2:**
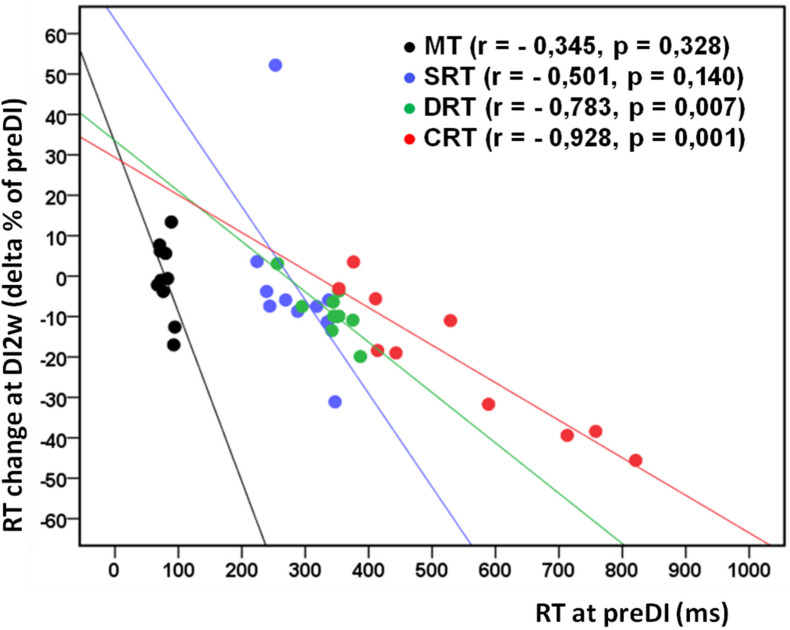
Individual change of the studied RT tasks (delta% of preDI) in the DI group (*n* = 10) at the study point “DI2w” (2 weeks after DI) in respect of the background data (point “preDI”), with correlation coefficients and approximation lines. MT, motor time; SRT, simple reaction time; DRT, discriminative reaction time; CRT, choice reaction time.

All subjects reported DI as a comfortable and relaxing procedure. Still, some subjects became worried about visiting the toilet by the end of the DI session. During DI, the systolic BP was stable, whereas the diastolic BP has decreased on average by some 6 mm Hg by the end of the DI session and heart rate by some 6 bpm, similar to the study of Meigal et al. (2018). Eight out of 10 subjects in the DI group fell asleep, usually by the 15th min of the DI session. The UPDRS-III, rigidity, and tremor scores did not change across the program of DI (Table 2). Before the program of DI, the studied RT tasks lined up from SRT thru DRT to CRT (Table 3). From the TT task, MT was estimated at 80 ms, and the tapping score was at 185 on average. The tapping score, MT, SRT, and SDT did not change across the DI program or after it. Instead, CRT and DRT have significantly decreased at the “DI2w” study point (Table 3). More specifically, at that point, CRT has on average lowered by 20% and DRT by 8% (Figure 1). Correspondingly, CPT has decreased by 50% (*p* < 0.05) and CFPT by 40% (*p* > 0.05). Thus, the mean percent of decrease of RT was greater in the tasks with longer initial response time. Improvement was more pronounced in patients with longer initial RT, as was statistically significant for CRT and DRT at the DI2w study point (Figure 2). Furthermore, this improvement did not correlate with levodopa equivalent dose (LED; *p* = 0.2). The initial UPDRS-III scores presented modest non-significant tendency to correlate only with the SRT improvement (*p* = 0.074). The accuracy of the studied RT tasks did not change across the program of DI (Table 3). In the noDI group, no significant change of the RT parameters was found, but the accuracy of reaction has significantly improved at the study point “DI2w” in SRT and DRT (Table 4 and Figure 1).

**TABLE 2:** Scores of the UPDRS indicators across the program of DI and after it in the DI group.

Parameters	PreDI	PostDI	DI2w	p*
UPRDS-III	23.4 ± 7.6	22.8 ± 7.0	21.7 ± 7.5	0.239
Rigidity subtotal	4.5 ± 2.9	4.6 ± 2.3	4.1 ± 2.4	0.589
Tremor subtotal	4.0 ± 3.1	3.6 ± 3.3	3.8 ± 2.9	0.287
Akinesis subtotal	9.1 ± 3.4	8.4 ± 3.3	8.1 ± 3.8	0.026

Data are mean ± SD. * Friedman’s test.

**TABLE 3:** Numeral characteristics of the RT tasks across the program of DI and after it in the DI group.

Parameters	PreDI	PostDI	DI2w	p*
Taps in the TT (N)	185 ± 24	197 ± 12	193 ± 21	0.154
MT (ms)	80.6 ± 9.5	76.7 ± 5.3	78.9 ± 9.5	0.452
SRT (ms)	284 ± 37	284 ± 43	290 ± 56	0.457
SDT (ms)	204.8 ± 45.8	196.2 ± 35.8	194.7 ± 49.5	0.717
Accuracy rate in SRT	0.97 ± 0.05	0.95 ± 0.06	0.98 ± 0.17	0.381
DRT (ms)	338 ± 38	329 ± 33	308 ± 24*0.011	0.007
CFPT (ms)	62 ± 47	48 ± 49	35 ± 44	0.119
Accuracy rate in DRT	0.936 ± 0.5	0.86 ± 0.17	0.943 ± 0.044	0.539
CRT (ms)	540 ± 162	490 ± 126	404 ± 49*0.011	0.014
CPT (ms)	255 ± 156	210 ± 106	130 ± 36 *p* = 0.057	0.037
Accuracy rate in CRT	0.917 ± 0.07	0.937 ± 0.08	0.967 ± 0.4	0.105

Data are mean ± SD. * Friedman’s test.

**TABLE 4:** Numeral characteristics of the RT tasks across the program of DI in the “noDI” group of PD subjects.

Parameters	PreDI	PostDI	DI2w	DI2m	p*
Taps (N)	200 ± 7	185 ± 19	193 ± 13	195 ± 17	0.323
MT (ms)	74.9 ± 2.7	82.0 ± 9.3	78.1 ± 5.1	77.4 ± 6.4	0.323
SRT (ms)	251 ± 52	234 ± 11	253 ± 13	257 ± 13	0.238
SDT (ms)	176 ± 50	152 ± 19	174 ± 10	180 ± 15	0.145
Accuracy rate in SRT	0.913 ± 0.07	0.953 ± 0.04	0.98 ± 0.02*	0.96 ± 0.06	0.037
DRT (ms)	300 ± 30	305 ± 31	311 ± 28	318 ± 30	0.896
CFPT (ms)	49 ± 59	72 ± 27	58 ± 32	61 ± 20	0.896
Accuracy rate in DRT	0.93 ± 0.03	0.98 ± 0.018	0.99 ± 0.015*	0.92 ± 0.03	0.005
CRT (ms)	411 ± 53	433 ± 49	423 ± 53	422 ± 43	0.724
CPT (ms)	159 ± 71	199 ± 42	171 ± 61	165 ± 39	0.166
Accuracy rate in CRT	0.90 ± 0.07	0.95 ± 0.06	0.97 ± 0.03	0.98 ± 0.03	0.190

Data are mean ± SD. * Friedman’s test.

## Discussion

The original hypothesis of this study held that (1) the ground-based μG modeled with DI would have improved the performance of the RT tasks in subjects with PD and (2) specifically in the tasks of higher cognitive demand. In line with these hypotheses, we found that RT has indeed improved by 20% in CRT, the most cognition-loaded task. In DRT, a less complex task, RT has decreased by 8%. In such least complex task, as SRT, RT has not changed. Thus, the more cognitively loaded was the task, the more notable was the effect of the DI program on it. Next, we found that the improvement of RT was associated only with the reduction of the non-motor part of RT, as MT did not change across the program of DI. Instead, such non-motor RT components as CPT and CFPT, which represent, respectively, time to choose between two stimuli and time needed to ignore stop-signals (attention), have decreased, though CFPT not significantly. Finally, the most profound decrease of RT was seen 2 weeks after the DI program, in accordance with our earlier findings for the clinical indicators of PD (Meigal et al., 2018). However, in the present study, clinical measures (UPDRS-III scores and subtotals of rigidity and tremor) did not significantly change. Partly, that discrepancy could be explained by smaller mean pre-treatment UPDRS-III scores in the present study—24 vs. 29 from the study of Meigal et al. (2018). Additionally, in the present study in four subjects, UPDRS-III did not change at all (subjects 5, 6, 9, and 10). The neurophysiologic, circulation, hormonal/neuroendocrine, behavioral, neural plasticity, and acclimation mechanisms may have contributed to such results.

### Neurophysiologic Mechanisms

The assumption that RT is modified under DI due to neurophysiologic mechanisms stems from the electroencephalographic (EEG) studies under various μG conditions. For example, the saccadic RT was reported to correlate with α-waves on EEG in the tasks for attention (Hamm et al., 2012). By this fact, it seems that attention and RT are improved when α-waves on EEG become more powerful. Indeed, suppression of β-wave bursts on EEG helped in decreasing RT (He et al., 2020). During the 5-day DI experiment, α-waves in young subjects were most powerful on the fifth day, supposedly due to reduced sensory flow to the brain cortex from the sole supportive skin zone (Lazarev et al., 2018). Similarly, α-waves on the EEG become more powerful after the fourth day of spaceflight that was attributed to the withdrawal of the vestibular gravity-related sensory signals (Van Ombergen et al., 2017). Missing gravity, induced with parabolic flight, clearly improved attention (Wollseiffen et al., 2016; Wollseiffen et al., 2019). These studies are supportive of the hypothesis that DI induces a kind of physiological “deafferentation” of proprioceptors that would have led to the reservation of vacate nervous circuits and compensatory hyperexcitability of the sensory pathways (Acket et al., 2018). We admit that in our study, sensory flow to the brain cortex was substantially decreased due to the reduction of signaling from proprioceptors, which presumably have improved RT specifically in such more complex tasks, as DRT and CRT. As for the vestibular sensory input, it had hardly contributed to the result as gravity is not eliminated in the DI model of μG.

### Cerebral Hemodynamics

It is well established that during either ground-modeled or on-board μG, fluids shift to the upper body and head (Tomilovskaya et al., 2019) that potentially could improve brain oxygen supply and, hence, better mental performance (Wollseiffen et al., 2019). However, the periods of μG in the study of Wollseiffen et al. (2019) were extremely short (around 20 s), and they alternated with the periods of hypergravity. Longer sessions of μG, either on-board or simulated, induce presumably an increase in intracranial pressure and decrease of carotid flow (Arbeille et al., 2017; Kermorgant et al., 2017), which would have hardly improved brain oxygen supply.

### Hormones and Humoral Mechanisms

Either real spaceflight or ground-based μG modifies neuroendocrine mechanisms and the concentration of hormones. For example, the endocannabinoid system becomes activated under parabolic flight (Strewe et al., 2012), and such hormones, as adrenocorticotropic hormone (ACTH) and cortisol, are increased under parabolic or spaceflight (Schneider et al., 2007). In PD subjects, water immersion in combination with exercises was reported to decrease the concentration of serum cytokines, thus exerting anti-inflammatory (Pochmann et al., 2018) and pro-antioxidative effects (Dani et al., 2020). In healthy people, brain-derived growth factor is increased after water immersion (Kojima et al., 2018). In sum, DI could have exerted a beneficial effect on the nervous activity of PD subjects by modification of humoral factors.

### Behavioral Factors

Several behavioral factors could have influenced on DI action on RT: (1) sleep behavior, (2) the “speed–accuracy tradeoff” (SAT) that refers to a strategy of choice between the speed of the RT task and its accuracy (Heitz, 2014), and (3) learning. Indeed, after one night of sleep deprivation, DRT, but not SRT or MT, has significantly increased (Skurvydas et al., 2020). In our earlier study, many of the subjects with PD slept better during the program of DI, and some of them fell asleep right upon immersion (Meigal et al., 2018), which was also the characteristic of the present study. This prompts that sleep behavior modification may, at least partly, be responsible for the beneficial action of DI on DRT and CRT. In comparison with younger healthy controls, older subjects and patients with PD normally rely on accuracy rather than on speed of RT (Wylie et al., 2009). Furthermore, RT in error attempts differs from those in correct (Huang et al., 2015). In the present study, no effect of DI on the accuracy of the RT tasks was found. Yet, we neither took SAT into account, because our subjects were not instructed to perform the RT tasks specifically accurate or fast. Still, we must report that in the second attempt, subjects with PD usually performed faster than in the first one, and for that reason, the second attempt was more often taken into analysis. So, we must admit modest interference of “speed” instruction, and hence, SAT, into the results of our study. In our future studies, SAT must be considered and studied. Finally, the absence of RT modification in the control group makes the contribution of learning to the result highly improbable. However, increased accuracy of reaction in the noDI group at the study point “2Wpost” suggested that learning still took some place.

### The “Warm Water” Effect and Acclimation

Some studies report on the “warm water” effect during aquatic therapy (Masiero et al., 2019). This effect implies mental and muscular relaxation characteristics for thermally comfortable warm environment. Therefore, the “warm water” effect may have contributed to the condition of functional “deafferentation” induced by DI. Consistent with Masiero et al. (2019), in our earlier study, we reported on better motor performance in PD patients under air exposure at 40°C (Meigal and Lupandin, 2005). Acclimation to specific environmental factors, e.g., cold or heat, hypoxia, or to physical exercise retains for some weeks after the end of the studied exposure. For example, adaptation to six 60-min hot water immersions has retained for 2 weeks (Zurawlew et al., 2019). The rate of acclimation decay to the studied factor was estimated at the level of 2.5% per day after the end of exposure, and it depends on the number of separate exposures (Daanen et al., 2018). This is in line with our result that the effect of DI on RT was still seen 2 weeks after the program of DI. However, this does not explain why the effect of DI was most significant 2 weeks after the program.

### Neural Plasticity and Brain Connectivity

The most significant effect of DI on RT in PD subjects was seen 2 weeks after the program of DI. This is in line with our earlier result that such clinometric estimates of PD as UPDRS-III, muscle rigidity, and depression scores had most significantly improved, namely, 2 weeks after the program of DI (Meigal et al., 2018). This prompts that, along with neurophysiologic reactions to DI, such slower longer-term morphological mechanism as neural plasticity comes into play under the DI program (Acket et al., 2018). In PD patients, neural plasticity in the form of modification of synaptic transmission and neurogenesis indeed takes place under exercise interventions (Petzinger et al., 2013) and deep brain stimulation (Jakobs et al., 2020). Brief water immersion facilitates neural plasticity seen as modification of brain cortical activity (Sato et al., 2019). Furthermore, brain connectivity, which in PD is decreased compared with healthy people (Hallett et al., 2020), programs RT in the cognition tasks (Zhang et al., 2019). After specific anti-PD therapies, e.g., deep brain stimulation, brain connectivity is improved (Mi et al., 2020). This allows suggesting that RT at the second week after the DI program has improved due to brain connectivity improvement.

## Conclusion

In conclusion, both research questions have got positive answer. First, the program of DI in PD subjects exerted a significant positive effect on the cognition-loaded RT tests, such as CRT and DRT. Second, the more is the RT task loaded with cognition, the more profound was the effect of DI program on it. Additionally, we found that the effect of DI was most notable and significant 2 weeks after the program. This result and literature survey suggest that the program of DI might have affected RT on several levels: (i) perceptual neurophysiologic level (with “functional deafferentation” mechanism), (ii) behavioral level (with better sleep), (iii) neuroendocrine level (with stress hormones and cytokines), and (iv) neuroplasticity level (with growth factors and modified brain connectivity). However, these suggestions are still to be experimentally verified. Furthermore, on the level of integrative organism, motion is probably less subjected to DI than cognition. Figuratively, though the brain (the head) itself was not immersed during DI, but only floating on the surface of the water, the mental functions have “attracted” more influence from DI than the body neuromuscular system.

### Limitations

There were several limitations to our study. First, the duration of the DI session was short (45 min) in comparison with common ground-based space experiments, which normally last for several days or weeks (Borovik et al., 2020). A longer DI session and/or more sessions in the program of DI would have shown a more prominent effect. The reason for such short DI session was that the subjects in our study were older people, and that they had PD. As such, most of them, even after considering all non-inclusion criteria, still had controlled arterial hypertension, impaired autonomous nervous regulation, and osteochondrosis. According to our experience, BP in PD subjects after a period of notable decrease has usually increased by the 60th min of DI. Some subjects with PD felt uncomfortable by the 45th min of DI due to the feeling of filled urinary bladder. Therefore, we restricted the duration of DI session to 45 min. The rationale to assign shorter DI sessions also stems from 45 to 60 min sessions of aquatic exercises for PD rehabilitation.

Some of the internationally used clinical evaluation scales, e.g., Mini-Mental State Examination (MMSE), Movement Disorder Society (MDS)-UPDRS, Montreal Cognitive Assessment (MoCA), State-Trait Anxiety Inventory (STAI), and Frontal Assessment Battery (FAB), are not in public property, so we abandoned searching for correlation between them and RT. Furthermore, preparation of the DI session and data collection was rather long (around 2 h) and exhaustive for PD subjects, which restricted conducting some time-consuming cognition tests. Finally, the sample size was small, because many of the potential subjects were not enrolled to the study due to the strict non-inclusion criteria, and some subjects were not available at the post-DI examination, which substantially reduced the database.

### Prospective

For better insight to the mechanisms of RT modification in PD subjects under DI, EEG-based methods (evoked potentials and brain connectivity) should be used. Then, evaluation of such cognition domains as execution, memory, attention, and timing would be helpful to study the effects of the DI program. Hemodynamic must be evaluated in PD subjects due to significant fluid shift during DI. Furthermore, the program of DI could be expanded beyond 7–10, or even more, sessions of DI to achieve a more profound effect on PD subjects. Finally, the results of this study have probably been affected by longer values of RT in PD subjects in comparison with young controls. Young controls have optimal RTs that are hardly improved with a program of short-term DI sessions. Nonetheless, young subjects must be enrolled in future studies.

## Data Availability Statement

The raw data supporting the conclusions of this article will be made available by the authors, without undue reservation.

## Ethics Statement

This study was reviewed and approved by the joint ethical committee of Ministry of Health Care of the Republic of Karelia and Petrozavodsk State University (Statement of Approval No. 31, 18.12.2014). The patients/participants provided their written informed consent to participate in this study.

## Author Contributions

AM contributed to the basic concept of the study, study design, and implementation (RT data collection and analyses); statistical analyses; interpretation of results; writing of the manuscript; and approval of the final draft. OT contributed to the study design and implementation (RT data collection and analyses), interpretation of results, and approval of the final draft. LG-M contributed to the study design and implementation (supervising the DI procedure; monitoring the ECG, blood pressure, and clinical condition of the subjects during DI; and data analyses), editing of the manuscript, and approval of the final draft. IS contributed to the basic concept of the study, study design, interpretation of results, and approval of the final draft. All authors contributed to the article and approved the submitted version.

## Conflict of Interest

The authors declare that the research was conducted in the absence of any commercial or financial relationships that could be construed as a potential conflict of interest.
